# Juicy June: a mass-participation snack-swap challenge—results from a mixed methods feasibility study

**DOI:** 10.1186/s40814-018-0310-8

**Published:** 2018-06-29

**Authors:** Dorota Juszczyk, Fiona Gillison

**Affiliations:** 1 Guy’s and St Thomas NHS Foundation Trust, c/o Occupational Health Service, St Thomas’ Hospital, Education Centre, Westminster Bridge Rd, London, SE1 7EH UK; 2 Department for Health, University of Bath, 1 West 4.107, Bath, BA2 7AY UK

**Keywords:** Obesity, Social marketing campaign, Healthy eating, Online intervention

## Abstract

**Background:**

Improving diet as a means of reducing the development of disease states and obesity is a public health priority. Although a growing number of countries have adopted policies to improve dietary patterns at the population level, as yet there are no established means of successfully bringing about change, suggesting that new approaches are needed. This study aimed to investigate the feasibility and proof of concept of a theoretically informed healthy eating intervention based on the model of successful month-long alcohol reduction or stop smoking campaigns (i.e. a mass-participation ‘challenge’ format).

**Methods:**

The study was a mixed methods feasibility trial and proof of concept of an online intervention. Adults were recruited to take part in a month-long ‘Juicy June’ challenge in which they nominated one unhealthy daily snack and committed to replace this with fruit or vegetables. Behaviour change techniques to promote motivation, increase self-efficacy, promote social support, self-regulation and habit formation were integrated into materials provided off- and online to support dietary change. A Facebook group was used to provide information, encouragement and foster social support. Diet quality was assessed before and after the intervention. Reasons for taking part, adherence to the snack swap, use of the intervention materials and experience of taking part were explored using quantitative and qualitative measures.

**Results:**

Ninety-one adults of whom 42% were either overweight or obese took part. Over the 4-week intervention period, participants consumed their intended fruit/vegetable snack on average 5 days/week; however, they still consumed their target unhealthy snack on average 2 days/week. Adherence to the snack swap was stable over the 4-week intervention period. The use of specific behaviour change tools (e.g. self-monitoring) was low. Sixty-seven percent of participants accessed the online Facebook forum, but there were no user-generated posts or content.

**Conclusions:**

The study demonstrated that the concept of a novel snack swap mass participation campaign is acceptable and feasible. Further piloting to explore how to promote greater engagement with men and ethnic minority groups and how to promote social support and maximise engagement with behaviour change techniques would be valuable.

## Background

It is estimated that worldwide, more than 1.9 billion adults are overweight, and of those over 600 million are obese [1]. Improvements in diet to reduce overall calorie intake and increase nutritional quality are a key part of any obesity policy [2]. A range of policies have so far been implemented including legislative change (e.g. sugar tax or fat tax), working with industry to reformulate processed foods [3], introducing nutritional information labelling, reducing food promotion (e.g. restrictions on television advertising of food and drink products high in fat, salt and sugar in or around programmes made for children), environmental restructuring [4], providing information and health promotion interventions (e.g. Change For Life, [5]) and providing behavioural support for individuals wanting to change [6, 7]. Although a growing number of countries have adopted policies to improve dietary and physical activity patterns at the population level, none have been sufficient to halt the rise in obesity prevalence worldwide suggesting new approaches for obesity prevention and treatment are needed.

Recent interest has developed around the use of self-directed care, facilitated online, given its large potential reach at relatively low cost [8, 9]. Online interventions have been shown to have small but meaningful beneficial effects [10], particularly when used in conjunction with some degree of personal interaction (e.g. alongside or following some one-to-one support), so may have good potential for further development [11, 12]. This study reports on the feasibility and proof of concept of one such self-directed intervention. On the basis that a high intake of energy-dense micronutrient-poor foods promotes unhealthy weight gain [13, 14], while increased intake of dietary fibre is protective against weight gain [15–17], we aimed to explore whether small dietary changes could be achieved through swapping an unhealthy snack for fruit or vegetables on a daily basis. Although there is no agreed definition of ‘snacks’, foods associated with snacking are usually energy dense, high in fat, low in protein and likely to promote over-consumption [18, 19]. Evidence suggests that weight gain could be prevented in approximately 90% of the population by the introduction of small dietary changes to reduce energy balance by 50 kcal/day [20], and further that 70% of the UK adult population consume fewer than the recommended five portions of fruit and vegetables a day [21], supporting this approach.

Self-directed care, facilitated online, has been successfully used in the alcohol and smoking domains, and one example is a ‘mass change attempt’ where individuals pledge to change their behaviour for a specified period of time (usually a month) (e.g. smoking cessation—Stoptober [22] and alcohol reduction, e.g. Dry January, [23, 24]). The core content of this type of intervention includes a novel name to attract attention, the provision of a simple message (i.e. stop drinking or smoking for a month), online delivery and the facilitation of social support (to help individuals manage the social context). Such campaigns have been successful in targeting at-risk populations and not only prompting experimentation with short-term behaviour change, but also leading to sustained effects. For example, the UK alcohol abstinence-based campaign Dry January showed that 1-month participation was associated with subsequent longer term reductions in alcohol consumption. The evaluation of Dry January, employing a prospective cohort study, showed that of 857 adults participating in the challenge, 64.1% successfully abstained from alcohol for 1 month. At 6 months follow-up, participation in Dry January was associated with drinking less in terms of the number and frequency of drinks and reducing the frequency of drunkenness [24]. However, participants with higher baseline alcohol intake were less likely to complete the 6-month follow-up; therefore, the results could be representative of those who drink less and have lower dependence scores, and as a result who have a better chance of succeeding in the challenge. Such campaigns remain largely untested in the dietary domain.

Recent research has identified the importance of incorporating theory into intervention design to maximise the efficacy of behavioural support [25, 26]. The specific content and delivery of this dietary intervention was therefore designed to be theoretically informed to address barriers and facilitators to dietary change identified in past work with the target population [27, 28] and evaluations of mass-participation health campaigns. Based on a review of past research, five factors were highlighted for targeting through our intervention: motivation, social support, self-regulation, self-efficacy and habit. These determinants were targeted both through the incorporation of relevant evidence-based behaviour change techniques (i.e. intervention content) and through the format of the intervention itself (i.e. a mass participation, month-long campaign).

We first consider intervention content: Motivation is a key factor underpinning whether or not people engage with opportunities to make healthy choices (e.g. improve their diet) [29], or respond with indifference (i.e. ignoring new opportunities) or reactance (i.e. further consolidating or exaggerating previous level of behaviour; [30]). According to Self Determination theory, behaviour is better maintained by autonomous as opposed to controlled motivation, that is, when the behaviour is considered to be personally meaningful, relevant and choicefully enacted rather than behaviour undertaken through feeling pressure or obligation [31]. There is a wealth of evidence to support the premise that health behaviours need to be autonomously motivated in order to be sustainable in the long term [32–34] and for the importance of autonomous motivation for healthy dietary choices in particular [35]. Autonomous motivation can be supported through the creation of a social environment that supports choice and ownership of decisions, uses non-controlling language, provides a meaningful rationale for change, and where possible provides fun and enjoyment [36]. As such, the intervention was designed to foster autonomy support in all materials and interactions.

Systematic reviews of dietary interventions consistently point to the primary importance of facilitating social support for change and using behaviour change techniques that promote self-regulation, such as self-monitoring and goal setting in successful interventions [6, 7]. These techniques were therefore also incorporated into the intervention design. Finally, recent work exploring how we better support the *maintenance* of changes in eating behaviours emphasises the role of habit [37, 38], which can be facilitated through implementation intentions (specific ‘if-then’ action plans attached to specific environmental cues).

Through selecting a mass participation, campaign-style event, we were also able to design-in factors providing social support, promote motivation and self-regulation as characteristics inherent within the intervention format. A common barrier reported by people trying to change their diet is feeling that they are not perceived as ‘normal’ , for example, leading to feeling stigmatised for violating expectations around snack sharing [27, 28]. Taking part in a mass-participation event may help people to explain one’s choices without feeling abnormal (participation in a range of such initiatives is now commonplace) and may normalise the behaviour if others in a person’s social network also happen to be taking part. The format provides a second benefit considering recent evidence that many people perceive leading a healthy lifestyle to be unrealistic as they lack the competence to enact all the necessary changes (e.g. get up early to exercise before breakfast etc.; [39]). This simple ‘snack swap’ intervention offers an opportunity for people to trial a single change in their eating behaviour for a pre-specified period of time in a supportive environment. Finally, by aligning the focus of the intervention with a daily cue (i.e. a specific point in the day or situation in which snacking routinely takes place), the intervention lends itself to habit formation (i.e. automation of the decision-making process).

The present study aimed to assess the feasibility and proof of concept of mass-participation healthy eating intervention to promote the initiation of dietary change through addressing the following objectives:

1. To record completeness of self-reported study measures and engagement with online resources (i.e. number of Juicy June Facebook page views and number of interactions) by the study participants.
2. To explore the level of adherence to the daily snack swap (intervention adherence).
3. To elicit the intervention participants’ views on the acceptability of
  - the intervention, including the frequency and content of contacts made as part of the intervention and
  - the evaluation process.

## Methods

### Design

The study was run as a mixed methods pre-post feasibility and a proof-of-concept trial of an intervention facilitated online.

### Participants

To mirror the marketing of existing month-long campaign-style health promotion interventions as mass participation events, a universal recruitment approach was taken to allow all potential participants to self-select whether or not the challenge would be meaningful to them. Participants had to be over 18, eat snacks considered unhealthy daily, have internet access at home and/or workplace and be fluent in English to take part. As this was a feasibility study for which formal power calculations are not appropriate, we planned to recruit all those who responded to the study adverts and were eligible into the intervention. To explore the qualitative questions, we aimed to recruit two focus groups of 4–8 participants, purposively seeking to include both those who completed the study and those who did not.

### Procedure

To mimic the approaches used in mass-change campaigns in tobacco and alcohol contexts, the whole intervention including the recruitment of participants was conducted online, and there was no face to face or verbal contact with participants. Adults were recruited through cost-free online (Facebook, Twitter, university online noticeboard) and local press outlets between mid-April 2013 and late May 2013. Upon recruitment (and prior to 1 June), participants were asked to provide baseline data and to complete dietary assessment.

### Intervention content and delivery

Behaviour change techniques (BCTs) were chosen to map to the key intervention targets of motivation, social support, self-regulation, self-efficacy and habit formation (Table 1).

**Table 1:** Behaviour change techniques as implemented within Juicy June

Behaviour change technique	Practical strategy
- 1.1 Goal setting (behaviour)/1.4 Action planning	- Participants prompted to decide specifically what snack to swap with what healthier alternative prior to the start.
- 2.2 Feedback on behaviour	- Dietary analysis provided following baseline measures in the format of a graph depicting personal intake alongside government guidelines using traffic light colour coding.
- 2.3. Self-monitoring of behaviour	- Hard-copies of self-monitoring sheets provided (to record each day snack-swap successfully achieved). Suggested to participants that this be attached to the fridge or other highly visible place.
- 4.1 Instruction on how to do a behaviour	- Clear information of what is intended by a ‘snack swap’, and what constitutes a healthy snack. Provided via email.
- 3.3 Non-specific social support	- General encouragement provided in standardised materials (including reminders to self-monitor). Weekly contact asking how participants had got on. - Enrolment on a Facebook group; regular encouraging posts uploaded onto the Facebook page every 2 days.
- 5.1 Information about health consequences	- Provided in study materials provided before and during the intervention
- 7.1. Prompts/cues	- Provision of a hard-copy calendar; weekly texts to cue preparation for snack swapping (e.g. shopping for target snacks)
- 7.2 Reduce prompts/cues	- Participants asked not to stock unhealthy snacks at home/in the workplace
- Promote autonomy support	- Study materials presented using non-controlling language (i.e. ‘you can, you may choose to’ rather than ‘you should, you must’); promotion of choice (in what aspects of diet to substitute); presentation of a rationale for change; provision of structure for behaviour change through outlining a simple snack-swap.

Notes: Behaviour change techniques are numbered according to the 93-item BCT taxonomy where included within this [62]. Autonomy support (aligned with Self-Determination Theory) is not listed within the taxonomy

#### Juicy June preparatory phase

Prior to the 1st of June (between 1 and 28 days depending on date of recruitment), participants received personalised dietary feedback on their daily intake of fruit and vegetables, fat and fibre and were encouraged to join the Facebook Juicy June community. Participants also received a Juicy June calendar to facilitate self-monitoring and completed a detailed plan (i.e. implementation intentions) for what snack swap they would attempt. On 30 May 2013 (2 days before the Juicy June launch) participants were sent final instructions to remind them of the steps involved in Juicy June.

#### Juicy June phase (1–30 June 2013)

On 1 June 2013, all participants were emailed to stimulate the start of the intervention trial. During the Juicy June challenge (i.e. between 1 and 30 June), regular Juicy June community updates were provided through Facebook, and participants were encouraged to seek and elicit social support via Facebook. At the end of each week, participants were sent an email asking them to report back on their goal attainment to assess their progress against Juicy June targets in the past week (they were sent four weekly evaluations in total). On 30 June 2013, participants received an email congratulating them on completing Juicy June and asking them to fill in the 4-week evaluation which was available online. Those who completed the final evaluation received a personalised dietary feedback comparing their baseline diet assessment and the 4-week evaluation assessment.

#### Post Juicy June phase (1 July 2013–1 August 2013)

All participants were invited to attend subsequent focus groups to report on their experience of taking part.

### Measures

#### Dietary assessment (used for dietary feedback)

Broad dietary questionnaires were used as a basis for exploring participant characteristics and providing feedback to participants on their diet, while the outcome measure for the trial was the report of weekly tally of healthy and unhealthy snacks consumed. Given challenges associated with measuring change in diet [40], a decision was made to specifically select a measure that reports on fruit and vegetable intake as it is likely to have the most sensitivity to this intervention. *Fruit and vegetable* intake was assessed by 24-h recall using a question adapted from the FACET (Five-a-day Community Evaluation Tool) questionnaire [41]. *Snack intake* was assessed using the Beverage and Snack Questionnaire [42], which rates consumption of 11 snack groups (e.g. crisps and popcorn, chocolate) over the past 7 days. Fibre and fat intake was assessed using the DINE (Dietary Intervention in Primary Care) questionnaire [41]. Participants were asked questions about six types of food categories they usually eat (bread; breakfast cereal; vegetable/fruit; foods high in fat such as cheese; type of milk; type of spread).

#### Goal achievement (outcome measure for the trial)

At the end of each week of the challenge, participants were sent a short online survey that aimed to measure adherence to the food swapping goals. Intake of unhealthy snacks was measured by one question: *How many days in the past week did you have your usual snack (food that you’re trying to avoid during Juicy June)?* assessed on a scale ranging from 0 to 7. Juicy June food intake was measured by one item: *How many days in the past week did you have your Juicy June food?* (response range 0 to 7).

#### Feasibility and acceptability

Participant engagement with the Juicy June Facebook page was quantified by analysing publicly available Facebook insights data (including usage data—unique page views which eliminate the factor of multiple views of the same page by the same user within a single session and active users) and total interactions (comments left by users, wall posts, total ‘likes’) [43].

Seven questions were used to capture participants’ experience of participation in Juicy June assessed on a five-point scale ranging from strongly disagree to strongly agree. These measured:

- Perceived difficulty of carrying out a snack swap
- Perceived difficulty of changing this behaviour as part of Juicy June compared with alone
- Whether participants felt like they were part of a Juicy June online ‘community’
- Frequency of conversations participants had about healthy eating with friends and family during Juicy June compared with usual
- Awareness of health benefits of eating fruit and vegetables
- Likelihood participants would attempt this or a similar swap in future

### Data analysis

Characteristics of participants were recorded at baseline and summarised through counts and percentages for categorical data and through means and standard deviations for continuous data. Outcome variables were summarised separately for baseline and follow-up using means and standard deviations for normally distributed continuous data. Post-intervention focus groups were planned to provide additional insight into the quantitative findings, exploring challenges encountered when trying to stick to a snack swap, experience of the Juicy June support package, and future intentions in relation to diet. Focus groups were digitally recorded and transcribed verbatim and analysed using thematic analysis [44]. The transcripts were coded by the first study author (DJ), clustered into themes, and to ensure trustworthiness, the themes that emerged from the data were discussed between the study authors (DJ and FG) to check that they were reasonably supported by the data. Given the limited number of people taking part in the focus groups, and restriction of the representativeness of participants involved, the focus group data was considered supplementary to the main analysis as information to assist in interpretation rather than generating new, generalizable data representative of study participants.

## Results

### Participants

One hundred twelve adults responded to the recruitment materials, of whom 91 enrolled and provided baseline assessment data (Table 2) and 61 (67%) completed the post-intervention follow-up. Participants’ mean age was 34.76 years (SD = 10.33), mean BMI at the baseline 25.75 (SD = 5.74) and 26.4% of the recruited sample were university students. Other baseline demographic characteristics and perceived health status are presented in Table 2. Data on goal achievement was provided by 85 participants at week one, 77 at week two, 78 at week three and 73 at week four. The most common reasons for taking part were to improve eating habits (76.7%), cut back on unhealthy foods (70%) and increase fruit and vegetable intake (68.3%) (participants could select more than one reason for their participation). Most participants in the discussion group had already been contemplating making dietary changes and saw Juicy June as a prompt to an already-intended action.

**Table 2:** Demographic characteristic and perceived health status of the sample (*N* = 91)

Variable	*N* (%)
Gender (% females)	78 (85.7)
Ethnicity (% White)	81 (89.0)
Employment (% full time)	66 (72.5)
Smoking status (% never smoked)	71 (78.0)
Special diet (% no special diet)	73 (80.2)
Health rating (% fair/good)	65 (71.7)
Weight self-perception (% somewhat overweight/very overweight)	61 (67.0)
Weight concern (% quite concerned/very concerned)	53 (58.1)
Weight harmful to health (% not at all harmful/not very harmful)	59 (64.8)
Weight control (% actively doing things to try to avoid gaining weight)	36 (39.6)

### Adherence

Seventy-nine participants chose to substitute their unhealthy snack for fruit (of those, nine chose dried fruit), three for a vegetable, and nine for either a fruit or a vegetable (i.e. their choice varied over time).

On average, participants consumed their proposed Juicy June fruit or vegetable on 5 days a week (M = 4.8, SD = .89, range = 2.5–6.5), while they consumed the snack they were trying to avoid on average 2 days per week (M = 2.0, SD = 1.18, range = 0–4.5).

### Engagement with materials and support

Twenty-two participants (24%) clicked at least once on the Juicy June Facebook page. Researcher-generated Facebook posts were viewed by between 0 and 16 unique participants, with each post being viewed between 23 and 119 times. There were no user-generated posts, and only one ‘like’.

### Participant experience (quantitative)

Of those providing data at post-intervention follow-up (*n* = 61), the majority (64%, *n* = 39) agreed that replacing an unhealthy eating habit with a healthier eating habit was easier with Juicy June than it would have been to do on their own. Approximately half (49%) of the participants found swapping habits difficult or very difficult, while 26% felt it was easy or very easy. Only 26% (*n* = 16) of participants felt part of a Juicy June online ‘community’ , although 43% (*n* = 26) reported having more conversations offline about healthy eating with friends and family during Juicy June than they did usually. Seventy-four percent (*n* = 45) of participants felt that they were more likely to consider making other similar swaps including fruit or vegetables in future, and 40% reported feeling more aware of the benefits of fruit and vegetables.

### Participant experience (qualitative)

Six participants (all female, aged 25–58 years old) who completed Juicy June took part in the focus group which was conducted on 10 July 2013. Pseudonyms have been chosen/selected by focus group participants themselves, and these do not represent identifying information. Recruitment of those who dropped out of the study was attempted but was unsuccessful. Perceived challenges to achieving the intended snack swap included difficulty in extinguishing well-established snacking habits, often eating these in addition to their healthy snack (Martha: *In my case it’s a habit. I might be on my own at home, but if I make a cup of tea or coffee I just have to, wait a minute, I’m missing something and have to have something with it*), the lack of appeal of fruit and vegetables compared with alternative snacks, preparation requirements and the ready availability of unhealthy snacks (Elizabeth: *I think also it just seems more interesting to have, like there is lots of different types of snack food, whereas if you buy fruit to make it interesting you sort of have to plan ahead and prepare it in a different way like make a fruit salad or bake it or do something with it to make it more interesting*); being offered unhealthy snacks (Amanda*: unfortunately I didn’t manage to sort of avoid (snacks) you know if it had been if I have been somewhere at the time of my morning coffee time I reckon I probably would have folded*). Challenges to engaging with the tools provided by Juicy June to support change included a perception of lack of personal usefulness of the tools, feeling awkward with interacting with a group of unknown people online (Sarah: *And that’s not normally the sort of thing I would put on Facebook. So I think that’s why I didn’t in the end. “Hey people I’ve eaten a banana” Who is interested?*) and not feeling self-monitoring was necessary (Amanda: *I had mine (Juicy June calendar) at home and… I thought it was a nice aid but I didn’t use it hugely and then I kind of guessed at the end of the week. Yeah I gave myself a tick.)*.

## Discussion

This study aimed to establish the feasibility and proof of concept of a theoretically informed mass-participation snack-swap intervention that aimed to prompt a small but potentially important change in dietary quality within the general population. The majority of participants (87%) chose to substitute their unhealthy snack for fruit. Data from the quantitative evaluation, supported through qualitative data collected from a sub-sample of participants, suggest that the concept of a novel snack swap mass participation campaign is acceptable and often very welcome to help them put their existing intentions to eat more healthily into action. Participants felt that introducing the snack swap was more difficult than expected and that taking part in a mass participation intervention was perceived to be helpful in this. According to weekly monitoring reports, participants consumed their proposed Juicy June fruit or vegetable on 5 days a week, while they consumed the snack they were trying to avoid on average 2 days per week. This pattern of consumption remained stable throughout the 4-week period, suggesting that the concept of a snack swap month-long intervention is acceptable and feasible. From the perspective of collecting research data, there was a relatively high level of attrition, as is common in online interventions [45]; completeness of post-intervention data was 67%. It is not known whether this was a result of participants losing interest in the intervention or simply not completing the follow-up questionnaire. The level of adherence and use of online resources was low, with less than 25% of participants having clicked at least once on the Juicy June Facebook page. No posts (such as comments) were generated by the Facebook group users. The results are similar to the result of other studies describing usages of social networking sites regarding topics of public health interest which suggest that while social media might be a good tool to rapidly disseminate information, it is more challenging to harness the full potential of social media for public health interventions (e.g. creating a persistent, stable network [46]). One possible reason for this low adherence to the Facebook group in the current study was that participants felt uneasy conversing via an online platform with strangers regarding their snack swap, as reported in the focus group. However, while participants were not interacting on Facebook, it appeared they engaged in offline social interaction as 43% of participants reported having more conversations about healthy eating with friends and family than they would normally have; therefore, it is possible that people felt more able to talk about the intervention and get support from their families as a result of taking part in a wider campaign. A national event, rather than small-scale research project, may also have greater draw to participants in engaging with online content.

While use of additional off-line tools was not measured across the whole sample, the results from the discussion group suggest that participants were not using the behaviour change tools provided and therefore did not receive the full intervention ‘dose’. For example, focus group participants did not widely use the self-monitoring calendar nor employ other methods of self-monitoring suggested (such as setting an email pop-up reminder). Thus, we cannot make firm conclusions as to what extent the behaviour change techniques employed in the current study accounted for the changes in snack consumption reported. Nonetheless, the completion of self-monitoring logs in dietary interventions has been found to be achievable in other studies (e.g. participants in a 24-week weight loss trial submitted an average of 14 of 24 self-monitoring diaries; [47]).

Juicy June aimed to communicate a relatively simple message and relatively achievable task. Recruitment resulted in a sample of relatively healthy weight and healthy eating individuals with high baseline autonomous motivation for weight control. This is an interesting finding itself as it suggests to whom the intervention was appealing. The aim of tobacco and alcohol mass change interventions was not only to engage those at risk, but also to create a mass change to normalise healthy behaviours and turn the campaign into a movement [48]. Thus, although Juicy June did not appear to attract those less motivated, it is possible that similar to the alcohol campaigns, once delivered yearly, the participation would increase through the contagion of social behaviour [49]. For example, participation in Australian Dry January increased from approximately 1000 participants in 2008 to over 18,000 in 2013 [50]. In a similar Australian alcohol reduction campaign, FebFast which began in 2007, by 2011, the majority of participants reported that they knew at least one person who had also participated, and one third participated along with their family members, friends or work colleagues [51]. Thus, it is perhaps the appeal to a sufficient number of people, rather than the inclusion of groups that we ideally want to reach, that is important at the initial stage of development of campaign-style interventions such as Juicy June.

### Limitations

This small-scale feasibility trial could not mimic the effects of a population-wide mass-participant event (which typically includes considerable marketing, media coverage and often affiliations with charities) and hence is restricted to exploring the acceptability and attractiveness of the concept. In particular, we acknowledge that the anticipated/presumed social support effects of a wider reaching campaign could not be simulated using the present study design. The accuracy of dietary measures also limits the reliability of all studies restricted to self-report measures which are subject to misremembering and desirability bias [52]. We attempted to increase recall and sensitivity of our primary outcome through two self-reported items asking about weekly frequency of consumption of a healthy and/or unhealthy snack at the times participants had nominated that their swap would take place (rather than a full food diary). However, we acknowledge that estimating the frequency of target snacks in this manner might not measure/elicit the mechanism by which snack swap intervention works (e.g. direct substitution, both snacks are eaten or otherwise).

While active engagement with the Facebook group was assessed by examining Facebook statistics, this approach is limited as an indicator of value and utility, as it reports on the number of unique people who have seen the post and the number of unique people who have clicked on a post, but does not indicate whether users really engaged. This may be better measured for example by measuring how much time viewers spent looking at a given post. In addition, within this pilot study, we did not measure whether greater Facebook use was associated with perceived social support or other benefits such as increased awareness of the benefits of a diet high in fruit and vegetables.

A final limitation was the study sample; we used an opportunistic sampling strategy that was not intended to be reflective on the general population and thus piloting on a wider group. The study recruited a sample which was 86% female suggesting that the intervention was particularly appealing to women. Given that interventions designed according to the characteristics of a sub-sample are unlikely to be effective across the whole population [53], including more men and different ethnic and social groups in the process of refining the intervention for future research is warranted.

Despite efforts to recruit to focus groups from across the participant groups, only one focus group could be recruited consisting of all women and no participants who dropped out from the study; therefore, we cannot generalise from this to the wider participant group. In part, this was due to the study being run online, so many participants were not within a reasonable travelling distance of the study site. Further, socio-economic status was not recorded within this study but could make a difference to the potential for intervention effects. Lower social and socioeconomic status (SES) is associated with poorer health behaviours and poorer health outcomes [54, 55]. For example, in the UK, only 19% of men and 23% of women in the lowest income quintile eat the recommended five portions of fruit and vegetables a day, compared with 30% of men and 35% of women in the highest income quintile [56]. It is likely that the current intervention would have different levels of feasibility and acceptability across different socioeconomic groups and have a different level of impact and efficacy on each. Mechanisms for this include the cost, perceived cost of healthy foods, levels of education and understanding and differing social norms and sources of support.

### Future research

The promising results of this feasibility study demonstrate that a month-long snack-swap campaign is acceptable and feasible and that the means of measuring its impact are sensitive to potential change. Given recommendations that a series of pilot studies can be useful in enabling the refinement of a study design [57], a pilot trial incorporating adaptations to the intervention is warranted [58]. Adaptations indicated by this feasibility trial that would be useful to incorporate in an updated pilot version include more usable self-monitoring tools (e.g. incorporating digital technologies), exploring other means of facilitating social support (e.g. recruiting whole workplaces/communities to take part together) and trailing a more sensitive measure of snack swap (e.g. daily mobile short messages service where participants are required to text back) which would help to elicit the mechanism by which the snack swap intervention might work. The use of a more sensitive measure of snack swap adherence would also help to explain whether participants who were unsuccessful had failed to adopt a new behaviour (eating an additional portion of fruits or vegetable) or failed to stop an old one.

Future research could also explore ‘what works for whom’ [59], by comparing the same intervention across different socioeconomic groups. The impact of a small improvement in diet may be greater in those with poorer starting levels. 42.6% of participants had more conversations about healthy eating with friends and family than they would normally have; therefore, a future study could appeal to a wider audience by encouraging participants to take part with someone they know (e.g. partner, friends, work colleague) as mobilising social support might be of key importance for such interventions. For example, a pilot study could be conducted within a workplace environment, where social norms and social networks associated with snack intake could be targeted; previous studies have shown the workplace to be a feasible setting for interventions to increase fruit consumption of office workers [60, 61].

## Conclusions

The aim of this study was to establish the feasibility of a novel theory-based intervention based on the model of successful month-long alcohol reduction or stop smoking campaigns and thus provide insight into the potential for adapting this approach to the dietary domain. The results suggest that encouraging participants to experiment with small and achievable dietary changes as a part of month-long snack swap is acceptable and feasible; however, more work is needed to increase adherence to the intervention tools.
